# From destination perceptions to visit behavior in Macao: a sequential mediation model of motivation, attitude, and intention among Mainland Chinese tourists

**DOI:** 10.3389/fspor.2026.1889131

**Published:** 2026-08-10

**Authors:** Fei Yang, Songyu Jiang

**Affiliations:** Rattanakosin International College of Creative Entrepreneurship, Rajamangala University of Technology Rattanakosin, Nakhon Pathom, Thailand

**Keywords:** mainland Chinese tourists, electronic word-of-mouth, Macao, perceived destination image, perceived destination value, visit motivation

## Abstract

**Introduction:**

This study examines how mainland Chinese tourists' destination-related perceptions are translated into self-reported visit behavior in Macao through a sequential psychological process. Drawing on destination image theory, perceived value theory, push–pull theory, and the Theory of Planned Behavior, the study develops a sequential mediation model linking perceived destination image, electronic word-of-mouth (eWOM), perceived destination value, visit motivation, visit attitude, visit intention, and visit behavior.

**Methods:**

A quantitative survey was conducted among mainland Chinese tourists who had previously visited Macao. A total of 693 valid responses were collected. SPSS was used to conduct descriptive statistics, reliability analysis. AMOS was employed for confirmatory factor analysis, structural equation modeling, and bootstrap mediation analysis.

**Results:**

Perceived destination image, eWOM, and perceived destination value were positively associated with visit motivation. Visit motivation was positively associated with visit attitude, which was positively associated with visit intention. Visit intention, in turn, was positively associated with self-reported visit behavior. Bootstrap analysis further supported the sequential mediating roles of visit motivation, visit attitude, and visit intention in linking destination-related perceptions to visit behavior.

**Discussion:**

The findings extend tourism behavior research beyond intention-centered explanations by demonstrating how destination image, online information, and value evaluations are converted into behavioral outcomes through a staged psychological mechanism. The results also indicate that Macao tourism stakeholders should develop a more diversified destination image, strengthen the credibility and usefulness of eWOM, and enhance tourists' perceived destination value to facilitate the conversion of favorable perceptions into visit behavior.

## 1 Introduction

Tourism has become an important driver of destination economic recovery, urban competitiveness, and service-sector development. In the post-pandemic period, destination competition has shifted from attracting visitor arrivals alone to rebuilding tourist confidence, improving perceived value, and converting favorable perceptions into actual travel behavior (1, 2). With the continued recovery and diversification of the global tourism market, destinations are no longer evaluated only through physical attractions, but also through tourists' perceptions, online information, emotional evaluations, value judgments, motivations, attitudes, intentions, and behavioral responses (3). Recent tourism research has also emphasized that tourist behavior is usually formed through a staged psychological process, in which cognitive evaluations and affective responses gradually develop into behavioral intention and action (4, 5).

As a Special Administrative Region of China, Macao is widely known for its integration of Chinese and Western cultures, world cultural heritage, integrated resorts, gastronomy, festivals, leisure tourism, and urban entertainment. At the policy level, Macao has been positioned as a “World Center of Tourism and Leisure,” and the local government has continued to promote the integration of “tourism +” with gastronomy, MICE, events, sports, culture, and other sectors to support economic diversification (6, 7). In 2024, Macao recorded more than 34.90 million visitor arrivals, recovering to 88.6% of the 2019 pre-pandemic level, while mainland Chinese visitors remained the largest source market (6). This indicates that Macao's tourism recovery is not only a matter of market volume, but also a matter of whether the destination can transform tourist perceptions and short-haul travel interest into deeper destination engagement and visit behavior.

Despite this recovery, Macao still faces several destination-management challenges that may weaken the conversion from tourist interest to actual visit behavior. Firstly, Macao's destination image has been closely associated with gaming and entertainment, while its cultural, culinary, heritage, festival, and leisure resources are not always fully recognized by tourists. Previous studies have shown that there remains a gap between Macao's diversified destination positioning and tourists' perceived image of the city (7, 8). Second, Macao is characterized by high tourism density, limited urban space, and a relatively short average length of stay. Official statistics show that the average length of stay of visitors in 2024 was only 1.2 days, suggesting that visitor growth does not necessarily result in longer stays or deeper destination participation. Third, the price and quality of the service, accessibility and other factors in the tourists' minds may affect their motivation and behavior. For mainland Chinese tourists, Macao is often a short-haul and cross-border destination, which makes value-for-money, time efficiency, travel convenience, and information reliability particularly important. Therefore, Macao's practical challenge is not simply attracting more visitors, but improving the psychological conversion from destination perception to motivation, attitude, intention, and behavior.

Electronic word-of-mouth (eWOM) has become especially important in this conversion process. Tourists increasingly rely on online reviews, travel platforms, social media posts, short videos, travel notes, and user-generated pictures when evaluating destinations. Recent studies have shown that eWOM can influence destination image, tourist confidence, travel intention, and destination choice, especially when travelers face uncertainty in the post-pandemic environment (9, 10). Positive eWOM can reduce perceived uncertainty, provide external confirmation, and strengthen tourists' confidence in visiting Macao, whereas negative eWOM related to crowding, high prices, poor service, or low value for money may weaken travel motivation and behavioral readiness. In addition, recent research on tourism information quality shows that user-generated and firm-generated information can shape destination image, destination trust, and behavioral intention (11). Therefore, in the Macao context, eWOM should not be treated merely as an information channel, but as a digital cue that interacts with destination image and perceived destination value before tourists form motivation and attitude.

From a theoretical perspective, the conversion of destination-related perceptions into tourist behavior requires a sequential explanation. Perceived destination image, eWOM, and perceived destination value represent three complementary forms of pre-visit appraisal. Perceived destination image reflects tourists' evaluation of Macao's attractiveness; eWOM provides external information confirmation and reduces travel uncertainty; perceived destination value captures tourists' cost-benefit evaluation of the trip. Together, these factors form the perceptual basis for visit motivation. Once motivation is activated, tourists are more likely to develop a favorable attitude toward visiting Macao, which further strengthens visit intention and supports visit behavior. This sequential logic is particularly important in the post-pandemic tourism environment because tourists may be more cautious about safety, service quality, price fairness, time cost, and experience value before making travel decisions. Recent tourism studies have increasingly recognized that destination image, information quality, and online communication affect tourist responses, but these studies often focus on intention rather than the full behavioral conversion process (5, 9, 11).

Although existing studies have provided useful insights into Macao tourism, several research gaps remain. Previous research has mainly focused on specific tourism sectors or themes, such as gaming tourism, casino hotels, integrated resorts, food-related tourism experiences, memorable tourism experiences, and smart tourism development (12). These studies offer some specific cases of the tourism industry in Macao. However, they provide limited explanation of Macao as an integrated tourism destination and insufficiently examine the psychological mechanism through which mainland Chinese tourists respond to Macao's overall destination image, online information environment, and perceived value. As a result, the destination-level behavioral conversion mechanism among mainland Chinese tourists remains underexplored.

In addition, much tourism behavior research continues to use travel intention or revisit intention as the final outcome, while actual or self-reported visit behavior receives less attention (13). However, intention does not always lead to behavior. Studies on tourist and consumer behavior have shown that individuals may express a willingness to travel, revisit, or participate in destination activities, but such intentions may not necessarily be transformed into actual behavioral engagement (14). This intention–behavior gap is particularly relevant to Macao because tourists may hold a positive attitude toward the destination but still limit their visit to short stays, shopping, gastronomy, or entertainment activities rather than deeper cultural and leisure experiences. Therefore, visit behavior should be examined as a distinct behavioral outcome rather than being inferred only from intention.

Furthermore, previous studies have confirmed that destination image, eWOM, and perceived value are related to tourists' attitudes, satisfaction, intentions, and behavior (15). Nevertheless, these variables have often been examined separately or through partial relationships. Less attention has been paid to their sequential interaction as front-end perceptual factors that activate visit motivation and subsequently shape attitude, intention, and behavior. This limitation weakens the theoretical explanation of tourist behavior formation because tourist behavior is usually not the result of a single direct effect. Rather, it develops through a multi-stage psychological process in which destination image provides attraction-based perception, eWOM offers external information confirmation, perceived destination value reflects cost–benefit evaluation, motivation activates the desire to visit, attitude evaluates the visit positively or negatively, intention expresses behavioral readiness, and visit behavior represents the final behavioral outcome. Therefore, a sequential mediation model is needed to explain the transformation from destination-related perceptions to tourist behavior in the Macao context.

Based on the above practical and theoretical gaps, this study investigates the sequential psychological mechanism through which perceived destination image, eWOM, and perceived destination value are converted into visit behavior among mainland Chinese tourists visiting Macao. Specifically, this study proposes that destination-related perceptions first stimulate visit motivation, which then influences visit attitude, visit intention, and ultimately visit behavior. By integrating destination image, digital information, perceived value, motivation, attitude, intention, and behavior into a single sequential framework, this study responds to the need for a more complete explanation of tourist behavior conversion in Macao's post-pandemic tourism environment. The research objectives are as follows::

RO1: To explore the influence of perceived destination image, eWOM and perceived destination value on the visit behavior of mainland Chinese tourists in Macao through a sequential psychological path.

RO2: To examine the mediating effects of visit motivation, visit attitude and visit intention in the path from destination-related perceptions to visit behavior.

## 2 Literature review

### 2.1 Theoretical foundation

In this study, the theory of planned behavior, push-pull theory, destination image theory, and perceived value theory are integrated into a sequential psychological framework that explains the transformation from destination-related perceptions to visit behavior. In this framework, destination image theory and perceived value theory explain the formation of tourists' pre-visit appraisals. eWOM represents an external digital information cue that confirms, strengthens, or weakens such appraisals. Push-pull theory explains the activation of visit motivation, while the theory of planned behavior explains the subsequent transition from attitude to intention and behavior. Therefore, the theoretical logic of this study follows a staged process in which perceived destination image, eWOM, and perceived destination value first shape visit motivation, visit motivation then influences visit attitude, visit attitude strengthens visit intention, and visit intention finally contributes to visit behavior.

Destination image theory explains how tourists form a general impression of a place through cognition and emotion. Cognitive images are the images in tourists' minds of the destinations, such as attractions, facilities, accessibility, culture and services. Affective image refers to the emotions tourists have about a place, such as whether they find the place pleasant, peaceful, fun or interesting, and so on (16). Previous studies have found that the destination image affects tourists' motivation, attitude, satisfaction, loyalty and behavior intention to some extent (17). Perceived destination image in this study refers to mainland Chinese tourists' cognitive and emotional evaluations of Macao as a tourist destination. Within this model, perceived destination image represents tourists' attraction-based appraisal of Macao, including cultural heritage, leisure facilities, entertainment venues, urban environment, service atmosphere, and emotional appeal. This appraisal is expected to provide the perceptual foundation for tourists' motivation to visit Macao.

Perceived Value Theory explains tourists' behavior as a result of value evaluation. Sánchez-Fernández and Bonillo (18) consider the consumer's overall judgment of a product or service based on the relative strength of its perceived advantages over perceived disadvantages to be perceived value. Perceived value in tourism research generally refers to the tourists' sense of whether the value of the destination experience justifies the cost (including money, time, effort and emotional investment) they have paid (3). Research by Rueda-López et al. (19) has shown that perceived value affects the satisfaction, loyalty and intention to revisit and behavior intention of tourists. Perceived destination value refers to all-around evaluations by mainland Chinese tourists of the functions and emotions they expect to gain from a visit to Macao in this study. In the framework, perceived destination value functions as a cost-benefit appraisal before attitude formation. Mainland Chinese tourists may evaluate whether a visit to Macao is worthwhile in terms of price, time efficiency, service quality, convenience, cultural experience, entertainment opportunities, and emotional enjoyment. Such value evaluation is expected to strengthen or weaken visit motivation before tourists form a clear attitude toward visiting Macao (20, 21).

Electronic word-of-mouth is introduced as a digital information cue that complements destination image and perceived value. Unlike perceived destination image, which reflects tourists' own evaluation of Macao's attractiveness, eWOM provides external confirmation through online reviews, travel notes, short videos, social media posts, and user-generated travel experiences. Previous studies have shown that user-generated content and eWOM are important sources of tourism information because tourists increasingly use online reviews, photos, videos, and social media narratives to evaluate destinations and make travel decisions (22). eWOM may reduce travel uncertainty, improve destination familiarity, and help tourists judge whether Macao is convenient, enjoyable, and worth visiting. Online reviews can reduce perceived risk in tourism and that eWOM is positively related to destination image and travel intention (23). Therefore, eWOM is positioned as a pre-visit information appraisal that influences visit motivation together with destination image and perceived destination value.

Push-pull theory offers the basis for the theory of visit motivation (24) classify tourists' behavior into two categories according to the above theory: internal motivators and external attractions. Push factors are tourists' inner desires for relaxation, escapism, novelty, learning and family time, and social interaction. Pull factors are external attractions for visitors, such as rich cultural and recreational resources, good food and dining options, entertainment activities, shopping facilities, attractions, etc. Earlier studies have shown that push and pull motives are the reasons why tourists choose a place for travel and undertake this trip (25). Visit motivation in this study refers to the inner and outer reasons for the visit of mainland Chinese tourists to Macao. More specifically, push factors in this study refer to mainland Chinese tourists' internal needs, such as relaxation, novelty seeking, cultural learning, short-break travel, family companionship, social interaction, and escape from daily routines. Pull factors refer to Macao's external destination attributes, such as world cultural heritage, gastronomy, shopping, festivals, entertainment, leisure facilities, integrated resorts, transportation convenience, and urban atmosphere. Perceived destination image reflects tourists' evaluation of these pull attributes; eWOM verifies or challenges these evaluations through digital information; and perceived destination value indicates whether the expected benefits of these pull attributes justify the costs of visiting. Therefore, Push-Pull Theory explains why these front-end appraisals are first converted into visit motivation rather than directly into behavior.

The Theory of Planned Behavior links attitudes and intentions to behavior. As Ajzen (26), proposed, people are more likely to act out of their positive attitude towards behavior and a desire for it. At present, many scholars have applied this theory in their research to explore tourists' travel intentions, repeated-visiting purposes, destination selections and behaviors. Nguyen et al. (10) have determined that electronic word-of-mouth (eWOM) promotes tourists' positive emotions about a place and their own sense of social norms, perception of self-control in travel behavior, and willingness to travel. Thus, online reviews and other forms of digital travel information can expand the explanation range of the TPB in tourism choice behavior. Based on previous studies, it is known that attitudes are directly related to the will to act, and such a will can also be observed. Visit attitude refers to the positive or negative assessment that mainland Chinese tourists have of visiting Macao; visit intention refers to their willingness or plan to visit Macao; visit behavior refers to the visiting actions or behavioral engagement with Macao as a tourism destination; and electronic word-of-mouth refers to mainland Chinese tourists' perceptions of the credibility, quality, quantity and completeness of online information about Macao. In this study, TPB is used to explain the later stage of the tourist decision-making process. However, the model extends the conventional attitude-intention-behavior sequence by placing visit motivation before visit attitude. This connection is theoretically important because motivation provides the psychological energy that makes tourists evaluate visiting Macao as favorable, meaningful, and desirable (27). In this way, Push-Pull Theory interfaces with TPB at the transition from motivation to attitude: destination-related pull appraisals activate motivation, and this motivation subsequently shapes tourists' evaluative attitude toward visiting Macao.

Accordingly, the theoretical foundation of this study can be summarized as an integrated sequential mechanism. Destination image theory explains the attraction-based perception of Macao; perceived value theory explains tourists' cost-benefit evaluation; eWOM explains the influence of digital information and external confirmation; Push-Pull Theory explains the activation of visit motivation through internal needs and external destination attributes; and TPB explains the transformation from attitude to intention and behavior. Together, these theories support the proposed model by explaining how perception develops into motivation, attitude, intention, and behavior, thereby providing the theoretical basis for the hypotheses developed below.

### 2.2 Research hypotheses and conceptual model construction

To respond to the sequential logic of the proposed model, the hypotheses are organized into three levels. The first level examines the direct effects among destination-related perceptions, visit motivation, visit attitude, visit intention, and visit behavior. The second level examines the single mediation effects of visit motivation, visit attitude, and visit intention. The third level examines the sequential mediation effects through which perceived destination image, eWOM, and perceived destination value are transformed into visit behavior. This hierarchical organization clarifies that H1–H6 test the basic structural paths, H7a–H9 test key psychological links, and H10a–H10c test the complete behavioral conversion mechanism.

#### 2.2.1 Direct effects among destination-related perceptions, motivation, attitude, intention, and behavior

Perceived destination image refers to tourists' cognitive and emotional perceptions of a place for tourists, encompassing the tourism resources and facilities in that area, the service environment, accessibility, cultural atmosphere and emotional appeal (28). The destination image function in the pre-visit decision-making stage is the tourists' view of how attractive a place is. A good image makes a place more attractive, meaningful and enjoyable for tourists, and thus increases their desire to visit; however, it does not directly result in a willingness to visit (29). Electronic word-of-mouth refers to online reviews, comments, recommendations, travel notes, short videos and other shared experiences of a place on digital platforms and social media (30). Destination images are tourists' own evaluations of the attractiveness of a place, and eWOM is external information that supports or contradicts this evaluation. Credible and useful general information online can reduce the anxiety of travel among tourists, make them feel more confident in selecting a place to go on holiday, and thus increase their wish to travel (31). According to recent tourism research, electronic word-of-mouth (eWOM) is influencing people's emotions towards a place and thus affects their intention to visit in the future (10). Therefore, in this way, eWOM serves as an information source for the motivation of tourists. Perceived destination value refers to a person's overall assessment of the benefits received from a place, taking into account the cost in terms of money, time, effort and emotions (14). Perceived Value in the pre-visit stage is a cost-benefit assessment. When tourists believe that they will gain various kinds of benefits during their trip to a certain place, such as functional purposes, emotions, culture and experiences, they will be more motivated to visit.

Together, the three components of tourists' pre-visit appraisal are: perceived destination image, electronic word-of-mouth, and perceived destination value. Perceived destination image is a reflection of how attractive people think a place is; electronic word-of-mouth serves to provide external verification; and perceived destination value refers to the cost-benefit analysis of visiting that place. Together, the three factors are responsible for a person's first impression. Motivation refers to the internal driving force that makes tourists wish to visit a place (28), so this pre-visit assessment is expected to first inspire the desire to travel. Based on the above, the following hypotheses are proposed.

H1: Perceived Destination Image positively affects the motivation to visit.
H2: Electronic word-of-mouth positively affects the motivation to visit.
H3: Destination Value Perceived by Tourists positively influences their desire to visit.

Motivation for visiting a place is an internal need or an external draw that inspires a person to travel there, and the attitude a person has towards travelling is their overall evaluation of such an experience (28). Push-and-pull theory proposes that stronger travel motivation can make tourists have a more positive psychological evaluation of the destination because motivation involves both individual needs and the perceived attractiveness of a place (24). Tourism studies have also previously found that the motivation for travel is positively correlated with one's attitude towards visiting and the actual willingness to do so (32). According to the Theory of Planned Behavior, an individual's attitude is required to have a behavior intention, and a positive attitude promotes the intention of tourists to behave (26). Tourism scholars have supported this view in their studies, and positively regarded destination attributes have been linked to increased travel intention and the wish to revisit. The same theory indicates that behavioral intention is close to behavior, and research on tourism has verified that a strong will to visit a place will be followed by more tourism-related actions (33). In light of the above, it is hoped that Macao tourists from the mainland with a stronger desire to visit will have more positive feelings, stronger travel intentions, and participate more actively in travel activities. Based on the above theory and evidence, the following hypotheses are proposed:

H4: Motives for a visit positively affect one's attitude towards that visit.
H5: Visit attitude positively influences the intention to visit.
H6: A higher visit intention is positively correlated with the actual behavior of visiting.

#### 2.2.2 Single mediation effects of motivation, attitude, and intention

The direct paths proposed above imply that motivation, attitude, and intention are not merely separate predictors, but sequential psychological links. Therefore, this study further examines whether these variables mediate the relationships between front-end perceptions and later behavioral outcomes.

Motivations for travel can provide psychological support to help tourists develop a positive perception of the destination. Destination Image Theory proposes that a good image of a destination can attract more tourists; according to the push-pull theory, this attraction can be converted into a strong desire to travel (16). Experimental research has also shown that the image of a tourist attraction is closely associated with a person's reasons for visiting, and these reasons in turn impact the experience tourists have when at the location (34). Electronic word-of-mouth will provide visitors with various online experience-based information; research by (35) indicates that eWOM can increase the demand and favorable attitude of travellers towards the destination. Perceived destination value can also motivate tourists to travel, as people are more inclined to have a positive evaluation if they believe that a place has sufficient functions and emotions (36). Therefore, under the conditions of Macao, perceived destination image, electronic word-of-mouth, and perceived destination value are expected to affect visit attitude through visit motivation. Based on the above theoretical and empirical support, the following hypotheses are proposed.

H7a: Visit motivation mediates the effect of perceived destination image on visit attitude.
H7b: Visit motivation is a mediator of the effect of electronic word-of-mouth on visit attitude.
H7c: Visit Motivation mediates the relationship between Perceived Destination Value and Visit Attitude.

Motivations for travel generally refer to the psychological reasons that prompt people to visit a particular place. Previous studies have shown that higher tourism motivation can improve the positive feelings and boost tourists' intentions to behave positively; that is, motivated tourists tend to see more meaning and desire in destination activities (28). Attitude is a type of antecedent for behavior in the Theory of Planned Behavior, and a positive attitude towards travelling will increase a person's desire to travel (26). Research on tourists' attitudes in recent years has also shown that different places for tourists have different levels of travel intention (37). Intention of visit is often considered to be the closest psychological predictor of tourist behavior. Bui (38) has expanded the Theory of Planned Behavior (TPB) to show that travel intention leads to behavior, and Zhou et al. (39) have also verified that behavioral intention enhances the connection between travel intention and subsequent tourist behaviour. Therefore, given the situation in Macao, mainland Chinese tourists with a stronger motivation to visit are more likely to have a positive attitude towards visiting; favorable attitudes further strengthen the desire to visit; and a stronger desire to visit leads to actual behavior. Based on the above theory and experiments, the following hypotheses are proposed:

H8: Visit attitude mediates the path from visit motivation to visit intention.
H9: Visit intention mediates the effect of visit attitude on visit behavior.

#### 2.2.3 Sequential mediation effects from destination-related perceptions to visit behavior

Tourist behavior is generally the result of a chain of psychological changes rather than a single direct cause. Destination image has been found to influence tourists' motivation, attitude, intention and future behavior; that is to say, through internal psychological responses, tourists' perception of a place can be transformed into behavioral outcomes (40). Electronic word-of-mouth (eWOM) is also a way to communicate; online reviews and shared travel experiences can reduce the risk tourists feel and improve their awareness of a place, thereby motivating more people to travel there (41). A high sense of the value of the place of vacation can inspire people to behave in a certain way and also provide some motivation for the behavior (14). According to the theory of planned behavior, attitudes and intentions are the necessary psychological links for behavior; at the same time, the push-pull theory has also shown that one's perception of a place of study can affect one's attitude (26). Therefore, in the context of Macao, perceived destination image, electronic word-of-mouth, and perceived destination value are expected to affect visit behavior through the sequential roles of visit motivation, visit attitude, and visit intention. Based on the above reasons, the following hypotheses are put forward:

H10a: Visit motivation, visit attitude and visit intention sequentially mediate the effect of perceived destination image on visit behavior.
H10b: Visit motivation, visit attitude and visit intention serially mediate the path from eWOM to visit behavior.
H10c: Visit motivation, visit attitude, and visit intention sequentially mediate the path from perceived destination value to visit behavior.

**Figure 1:**
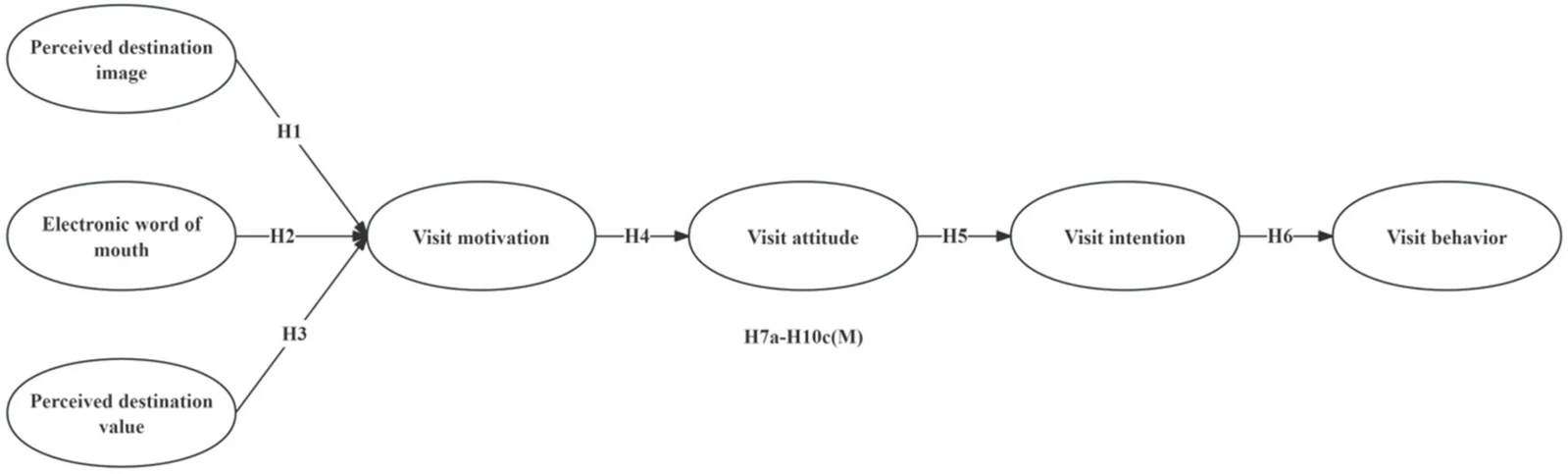
Integrated conceptual model for visit behavior.

Figure 1 presents the integrated conceptual model of this study. The model is structured around a sequential behavioral conversion logic consisting of three stages. The front-end stage includes perceived destination image, electronic word of mouth, and perceived destination value, which represent tourists' appraisal of destination attractiveness, external information confirmation, and cost–benefit evaluation. The middle stage includes visit motivation, visit attitude, and visit intention, which explain the psychological process through which destination-related appraisals are transformed into behavioral readiness. The back-end stage is visit behavior, which represents tourists' destination-related actions toward Macao. Specifically, the model proposes that perceived destination image, electronic word of mouth, and perceived destination value first influence visit motivation. Visit motivation then shapes visit attitude, which further strengthens visit intention and ultimately leads to visit behavior. Therefore, the model not only tests the direct paths from H1 to H6, but also examines the mediating and chain-mediating effects from H7a to H10c. This structure demonstrates that the four theories used in this study are connected through a sequential logic in which destination image theory, perceived value theory, and eWOM explain pre-visit appraisal; Push-Pull Theory explains motivational activation; and TPB explains the attitude-intention-behavior conversion. This integrated framework provides a concise explanation of the process through which mainland Chinese tourists' perceptions of Macao are converted into visit behavior through motivation, attitude, and intention.

## 3 Method

### 3.1 Data collection

A quantitative survey was conducted in this study, and data were collected from 693 mainland Chinese tourists who had visited Macao using a quota-based convenience sampling approach. The chosen target population is mainland China, the main source market for Macao tourism in China. In 2024, mainland Chinese tourists made up 70.1% of all visitors to Macao, and the top ten source regions were Guangdong, Shanghai, Zhejiang, Jiangsu, Hunan, Fujian, Hubei, Guangxi, Beijing, and Sichuan. These areas accounted for approximately 77.41% of the mainland Chinese visitors to Macao and were thus suitable for building the sampling frame of this study (6).

In practice, the quota-based convenience sampling approach was implemented through regional quota control and convenience-based respondent recruitment. First, quota references were established according to the ten major mainland Chinese source regions for Macao tourism, namely Guangdong, Shanghai, Zhejiang, Jiangsu, Hunan, Fujian, Hubei, Guangxi, Beijing, and Sichuan. The final valid sample included 252 respondents from Guangdong, 54 from Shanghai, 58 from Zhejiang, 60 from Jiangsu, 48 from Hunan, 55 from Fujian, 44 from Hubei, 50 from Guangxi, 34 from Beijing, and 38 from Sichuan, making a total of 693 valid responses. The purpose of setting regional quotas was to ensure that the sample covered the major mainland source markets and avoided excessive concentration in a single province or city. Data were collected through both online and offline channels. Online questionnaires were distributed via Wenjuanxing, WeChat, Xiaohongshu, Douyin, travel forums, blogs, email and other social media platforms. Offline survey points were set up in the selected cities of the ten main source areas and at representative places in Macao, such as commercial areas, tourism exhibitions, transportation hubs and popular tourist spots. A QR code is available to help the people fill out the questionnaire. Among the 693 valid responses, 470 questionnaires were collected online, accounting for 67.8% of the valid sample, while 223 questionnaires were collected offline, accounting for 32.2%.

To reduce the potential bias caused by convenience sampling, several procedural controls were adopted. First, quotas were set according to the major mainland Chinese source regions for Macao tourism to avoid excessive concentration in a single province or city. The final sample covered all ten selected source regions, which improved the regional diversity of the respondents. Second, both online and offline channels were used to reduce platform-specific bias. Third, two screening questions were included at the beginning of the questionnaire. The first question confirmed whether the respondent had previously visited Macao, and the second identified whether the respondent came from mainland China. Fourth, invalid questionnaires were excluded if they showed duplicate responses, extremely short completion time, patterned answering, or substantial missing values. For example, questionnaires were removed when respondents selected the same option for almost all scale items, failed to answer the screening questions correctly, or completed the questionnaire in an unrealistically short time. These procedures were used to improve sample relevance and response quality.

### 3.2 Measurement instruments and scale development

The measurement instrument was developed by adapting validated scales from previous tourism studies to the context of mainland Chinese tourists visiting Macao. The seven factors in the questionnaire are: perceived destination image, perceived destination value, electronic word-of-mouth, visit motivation, visit attitude, visit intention, and visit behavior. All the items were scored on a scale of 1–7, ranging from 1 = strongly disagree to 7 = strongly agree. The wording of the items was adjusted to fit the Macao tourism context while maintaining the original theoretical meanings of the scales,

To improve the transparency of the item-screening process, pilot reliability analysis was conducted before the formal survey. Items were considered for deletion when their corrected item-total correlation was below 0.40, Cronbach's alpha increased after item deletion, and the item showed weak conceptual consistency. Based on these criteria, four perceived destination image items (PDI12, PDI14, PDI17, and PDI18) and three electronic word-of-mouth items (EWM2, EWM9, and EWM10) were removed. Consequently, the Cronbach's alpha of perceived destination image increased from 0.902 to 0.927, while that of electronic word-of-mouth increased from 0.861 to 0.898. The first set of questions had 53 items, and after the reliability check, seven of them were discarded; thus, only 46 remain for the following analysis.

Because all variables were measured using self-reported questionnaire data from the same respondents, Harman's single-factor test was conducted to assess the potential influence of common method bias. All retained measurement items were entered into an unrotated exploratory factor analysis in SPSS. The results showed that the first unrotated factor accounted for 31.42% of the total variance, which was below the recommended threshold of 50%. Therefore, common method bias was not considered a serious threat to the validity of the results in this study.

Perceived destination image refers to the tourists' cognitive and emotional evaluations of Macao as a tourism destination. The two components of the construct were cognitive image and affective image. Almeida García et al. (28) and Jinquan et al. (36) adapted the original scale to create 18 items covering Macao's world cultural heritage, urban landscape, cuisine, lifestyle, cultural activities, nightlife, transportation, shopping, accommodation, public security, sanitation, vitality, modernity, and emotional experiences such as fun, relaxation, excitement, and pleasantness.

Perceived destination value refers to a person's general judgment of how valuable a trip to Macao is in terms of money, time, effort, and all-round benefits. The two indicators were function value and emotional value. Abbasi et al. (14) proposed a four-item scale to assess whether a trip to Macao was “worth it” based on money, time, effort and overall value, which has been adapted.

Electronic word-of-mouth is a person's perception of Macao, based on online travel reviews, comments and other information shared on digital platforms. The four indicators in the construction are: credibility, quality, quantity and completeness of electronic word-of-mouth. Anubha and Shome (30) developed a 12-item scale for knowledge and trustworthiness of reviewers, richness, helpfulness, persuasiveness, value of online reviews, quantity of online travel information, and completeness of information on Macao tourism, and adapted it for this study.

Motivation for travel refers to the internal needs and external attractions in destinations that motivate people to travel. The two are pushed and pulled motivations, respectively. The seven items of this scale were adapted from Almeida García et al. (28) and cover relaxation, cultural learning, adventure, excitement, shopping, entertainment, socialization, visits to friends or family, festivals, sports events, cultural performances, prestige, meetings, seminars and business activities.

Attitude of visitation is a general positive or negative assessment of the visit to Macao. Kara (42) developed a scale for tourism attraction evaluation that includes five semantic-differential indicators of visitors' attitudes towards sites in Macao Tourism, such as favourable-unfavourable, like-dislike, good-bad, pleasant-unpleasant and positive-negative. Visit intention is the tourist's willingness and plan to visit Macao. Jinquan et al. (36) provided four items from Jinquan et al. to measure this construct unidimensionally: attractiveness of visiting Macao, intention to visit under favourable circumstances, willingness to recommend Macao, and plan to visit Macao next year.

Visit behaviour refers to tourists' own-reported behaviourally engaged experience of visiting Macao, rather than just whether they have been to Macao. Al-Towfiq et al. (43) created the three items for an all-in-one index of this factor. The items measured how often respondents participated in Macao's tourism activities, such as visiting tourist attractions and cultural heritage sites, exploring destination resources, shopping, eating and drinking, leisure and entertainment during their trip, etc.

### 3.3 Data analysis

The data were analyzed using SPSS and AMOS according to their respective statistical functions. SPSS was used for descriptive statistics, reliability analysis, corrected item-total correlation analysis, KMO and Bartlett's tests, and Harman's single-factor test. AMOS was used for confirmatory factor analysis, structural equation modeling, and bootstrap mediation analysis.

Describe the demographic information of the subjects and show the distribution of key research items in the study. Frequencies and percentages were used for categorical variables, and means and standard deviations were employed to present the central tendency and spread of the data in the measurement items. The previous step obtained some basic knowledge about the characteristics of the sample and prepared the data for further inference. Reliability and Validity Analysis of the Measurement Scales have been Conducted. Cronbach's alpha was used to assess the internal consistency of each factor, and a value exceeding 0.70 was considered suitable. After deleting the item, the corrected item-total correlation and Cronbach's alpha were also checked to determine whether this item had poor measurement performance. KMO and Bartlett's test were used to determine whether the data met the conditions for factor analysis. A KMO value of 0.70 or higher and a statistically significant Bartlett's test indicate that the data are suitable for further validity testing. Use AMOS for confirmatory factor analysis to conduct a measurement-model test. Standardised factor loadings, composite reliability and average variance extracted were employed to assess convergent validity. Composite reliability values exceeding 0.70 and AVE values exceeding 0.50 were considered to meet the criteria for good convergent validity. Fornell-Larcker criterion for discriminant validity: Compare the square root of AVE with the correlation of the latent variables. Structural Equation Modeling (SEM) was employed in AMOS to test the proposed hypotheses and the structural relationships among perceived destination image, electronic word-of-mouth, perceived destination value, visit motivation, visit attitude, visit intention, and visit behaviour. Commonly used indices for model fit were employed, such as, RMSEA, GFI, AGFI, IFI, TLI and CFI. Standardised path coefficients, critical ratios and significance values were used to determine the direct effect. bootstrap was used to perform mediation analysis, and a 95% bias-corrected confidence interval was used. A mediator is positively correlated with either the independent variable or the end variable. Thus, the study was able to explore the direct path and the chain-mediated effect in the proposed conceptual model.

## 4 Results

### 4.1 Descriptive statistics analysis

Table 1 shows the basic information and tourism activities of the 693 respondents. The sample was mainly young and middle-aged mainland Chinese tourists, and among the respondents, those aged 25–44 made up 68.10% of the total. This age structure suggests that the respondents were largely active working-age tourists with relatively strong mobility, digital information exposure, and short-haul travel demand.

**Table 1:** Participants' demographics.

Variable	Category	Frequency	Percentage
Age	18 years old and below	7	1.00%
	18–24 years old	106	15.30%
	25–34 years old	294	42.40%
	35–44 years old	178	25.70%
	45–54 years old	78	11.30%
	55–64 years old	24	3.50%
	65 years old and above	6	0.90%
Gender	Male	325	46.90%
	Female	368	53.10%
Province/City	Guangdong	252	36.40%
	Shanghai	54	7.80%
	Zhejiang	58	8.40%
	Jiangsu	60	8.70%
	Hunan	48	6.90%
	Fujian	55	7.90%
	Hubei	44	6.30%
	Guangxi	50	7.20%
	Beijing	34	4.90%
	Sichuan	38	5.50%
Education	Junior high school or below	12	1.70%
	High school/technical secondary school	69	10.00%
	Associate degree	120	17.30%
	Bachelor's degree	340	49.10%
	Master's degree or above	152	21.90%
Marital status	Single	281	40.50%
	Married	41	5.90%
	Married with no children	112	16.20%
	Married with children	259	37.40%
Occupation	Student	73	10.50%
	Corporate employee	294	42.40%
	Civil servant	57	8.20%
	Freelancer	78	11.30%
	Professional technician	66	9.50%
	Service industry worker	49	7.10%
	Self-employed	60	8.70%
	Retired	16	2.30%
Monthly income	Less than 3,000 RMB	80	11.50%
	3,000–5,999 RMB	155	22.40%
	6,000–9,999 RMB	227	32.80%
	10,000–14,999 RMB	158	22.80%
	15,000 RMB and above	73	10.50%
Tourism activity type	Gastronomy and shopping tourism	526	75.90%
	Cultural and heritage experience tourism	472	68.10%
	Leisure and entertainment tourism	389	56.10%
	Urban short-break and social tourism	336	48.50%
	MICE and business tourism	78	11.30%

The proportion of women and men was about 53.10% and 46.90%, respectively. In terms of place of origin, Guangdong had the highest proportion at 36.40%, and respondents from Shanghai, Zhejiang, Jiangsu, Hunan, Fujian, Hubei, Guangxi, Beijing, and Sichuan were distributed among the various source markets for Macao tourism in other areas. The high proportion of Guangdong respondents reflects Macao's geographical proximity to the Guangdong-Hong Kong-Macao Greater Bay Area and the convenience of cross-border travel.

Most of the people in the sample were well-educated, and 71.00% had obtained a university degree or higher. This means that 71.00% of the respondents had received higher education, suggesting that many respondents were capable of evaluating destination information, comparing travel value, and using online travel platforms or social media content when planning visits to Macao. In terms of occupation, corporate employees represented the largest group at 42.40%, followed by freelancers, students, professional technicians, self-employed respondents, civil servants, service industry workers, and retired respondents. This occupational structure further indicates that the sample mainly consisted of economically active tourists who may have both consumption capacity and demand for short leisure trips.

Monthly income was mainly concentrated in the middle-income range. Respondents earning RMB 6,000–9,999 accounted for 32.80%, while those earning RMB 10,000–14,999 accounted for 22.80%. Together, these two groups represented 55.60% of the sample, showing that most respondents had moderate and relatively stable consumption capacity. For mainland Chinese tourists, especially those considering Macao as a short-haul cross-border destination, value-for-money may directly influence whether destination perceptions can be converted into visit motivation and behavior. Among the types of tourism activities, gastronomy and shopping tourism were listed as the most frequent, followed by cultural and heritage experience tourism, leisure and entertainment tourism, and urban short-break and social tourism. MICE and business tourism made up a relatively small part. These results suggest that mainland Chinese tourists' visits to Macao are not limited to gaming or entertainment. Instead, their activities are strongly associated with food, shopping, cultural heritage, leisure, and short-break experiences. As this item allowed for multiple answers, the percentages of the types of tourism activities shown do not sum up to 100 per cent.

Table 2 shows the descriptive statistics of the main factors. The average scores of all the constructs were above the midpoint of 4 in a seven-point Likert scale, so most respondents had a positive view and behaviour towards visiting Macao. Visit intention had the highest mean score (M = 5.124, SD = 1.054), followed by visit motivation (M = 5.104, SD = 1.000), perceived destination value (M = 5.037, SD = 1.125), and perceived destination image (M = 4.984, SD = 0.972). The above data show that most people believe Macao is a good place for tourists to visit and have a relatively strong will to travel there. Electronic word-of-mouth had the lowest mean score (M = 4.597, SD = 0.933), and although it was considered relatively positive, its credibility, utility, depth and coverage were all relatively weak.

**Table 2:** Descriptive statistics.

Construct	Number of items	Mean	SD	Minimum	Maximum
Perceived destination image	14	4.984	0.972	2.214	7.000
Perceived destination value	4	5.037	1.125	1.750	7.000
Electronic word of mouth	9	4.597	0.933	1.889	6.889
Visit motivation	7	5.104	1.000	2.286	7.000
Visit attitude	5	4.693	0.949	1.400	6.800
Visit intention	4	5.124	1.054	1.500	7.000
Visit behavior	3	4.858	1.044	1.667	7.000

### 4.2 Reliability analysis

Table 3 presents the reliability analysis results for the seven study variables. Cronbach's alpha was used to assess the internal consistency of each construct. In general, a Cronbach's alpha value above 0.70 indicates acceptable reliability, while values above 0.80 suggest good internal consistency. The results show that all constructs achieved acceptable or good reliability. The Cronbach's alpha values ranged from 0.815 to 0.941, all exceeding the recommended threshold of 0.70. Specifically, perceived destination image showed the highest reliability with a Cronbach's alpha value of 0.941, followed by electronic word-of-mouth with 0.916 and visit motivation with 0.894. Perceived destination value also demonstrated good reliability with a Cronbach's alpha value of 0.873. In addition, visit attitude, visit intention, and visit behavior had Cronbach's alpha values of 0.852, 0.816, and 0.815, respectively, indicating that these constructs also had satisfactory internal consistency. Overall, the reliability results indicate that the retained measurement items were stable and internally consistent. Therefore, all seven constructs were suitable for subsequent validity analysis and structural equation modeling.

**Table 3:** Reliability analysis.

Study variables	Number of questions	Cronbach's *α*
Perceived destination image	14	0.941
Perceived destination value	4	0.873
Electronic word of mouth	9	0.916
Visit motivation	7	0.894
Visit attitude	5	0.852
Visit intention	4	0.816
Visit behavior	3	0.815

Table 4 shows the results of the KMO and Bartlett's tests for suitability of data in factor analysis. The KMO value was 0.925, which exceeds the recommended value of 0.700; thus, the sample was considered to have a good sampling adequacy and met the necessary conditions for factor extraction based on correlation. Bartlett's test for sphericity was also significant; the approximate chi-square value was 18,436.572, degrees of freedom (df) = 1,035, and *p* = 0.000. The above results indicate that the correlation matrix is not the identity matrix and that some of the variables are correlated with each other. Therefore, the data were suitable for the following validity and confirmatory factor analyses.

**Table 4:** KMO and Bartlett's test.

Test	Index	Result
Kaiser-Meyer-Olkin Measure of Sampling Adequacy		0.925
Bartlett's Test of Sphericity	Approx. Chi-Square	18,436.572
	df	1,035
	Sig.	0.000

### 4.3 Confirmatory factor analysis

Table 5 shows the convergent validity results of the seven latent variables in the measurement model, including composite reliability (CR) and average variance extracted (AVE). All the convergent validity results indicate that the constructs have met the required measurement standards. The CR values were between 0.817 and 0.942; all of them exceeded the standard limit of 0.700, and thus indicated that the items in each factor had good internal consistency. All of the AVE values were above the recommended limit of 0.500, from a minimum of 0.529 to a maximum of 0.639; thus, all four constructs were deemed to have acceptable measurement reliability. Among the above indicators, perceived destination image had a coefficient of determination (CR) of 0.942, and perceived destination value had the highest average variance extracted (AVE) value of 0.639; both were deemed reliable and theoretically significant. These results indicate that the retained items had adequate internal consistency and explained sufficient variance in their corresponding latent constructs. Therefore, the constructs of perceived destination image, perceived destination value, electronic word-of-mouth, visit motivation, visit attitude, visit intention, and visit behavior all met the requirements for convergent validity.

**Table 5:** Convergent validity.

Variable	CR	AVE
Perceived destination image	0.942	0.540
Perceived destination value	0.875	0.639
Electronic word of mouth	0.918	0.556
Visit motivation	0.897	0.554
Visit attitude	0.857	0.548
Visit intention	0.817	0.529
Visit behavior	0.821	0.606

Table 6 shows the discriminant validity results according to the Fornell-Larcker criterion. The square roots of the AVEs for each construct are the diagonal elements, ranging from 0.727 to 0.799, and the off-diagonal elements are the correlations among the latent variables. Based on the above results, the square root of AVE for each construct exceeds its correlations with other constructs. For example, the square root of AVE for perceived destination image was 0.735, and this value was higher than its correlations with perceived destination value (0.367), electronic word-of-mouth (0.226), visit motivation (0.638), visit attitude (0.319), visit intention (0.392), and visit behaviour (0.375). The square root of AVE for visit behaviour was also 0.779 and was greater than its correlations with all other constructs. This indicates that each construct captured a conceptually distinct aspect of the proposed model. Therefore, the measurement model demonstrated acceptable discriminant validity and was suitable for testing the structural relationships.

**Table 6:** Discriminant validity.

Construct	1	2	3	4	5	6	7
Perceived destination image	**0** **.** **735**						
Perceived destination value	0.367	**0**.**799**					
Electronic word of mouth	0.226	0.203	**0**.**746**				
Visit motivation	0.638	0.468	0.526	**0**.**744**			
Visit attitude	0.319	0.381	0.332	0.515	**0**.**740**		
Visit intention	0.392	0.292	0.330	0.514	0.550	**0**.**727**	
Visit behavior	0.375	0.439	0.305	0.504	0.548	0.654	**0.779**

The diagonal is the square root of the AVE.

Bold values on the diagonal represent the square roots of the average variance extracted (AVE) for the respective constructs. Off-diagonal values represent the correlations between constructs.

Table 7 shows the good-of-fit statistics of the CFA measurement model. The value of *χ*2/df was 2.086, which was less than the upper limit of 3.000, and thus a good model fit was achieved. The RMSEA was 0.040, which is below the typical threshold of 0.080; thus, it can be considered to fit well. In terms of absolute fit indices, GFI was 0.887 and AGFI was 0.873; both met the requirement of 0.800 or more. The incremental fit indices were also as follows: CFI = 0.942, TLI = 0.939, and IFI = 0.943; all exceeded the recommended value of 0.900. Overall, the above results show that the measurement model has achieved an acceptable-to-good level of fit and is suitable for structural model analysis.

**Table 7:** Measure model fit statistics.

Fit index	*χ*2/df	RMSEA	GFI	AGFI	NFI	TLI	CFI	IFI
Reference standards	<3	<0.08	>0.85	>0.85	>0.85	>0.85	>0.85	>0.85
Result	2.086	0.04	0.887	0.873	0.905	0.939	0.942	0.943

### 4.4 Structural equation modeling (AMOS)

Table 8 shows the general fit results of the structural equation model. The results show that the value of $\chi^2$/df was 2.154, which is less than the recommended limit of 3; thus, the model is considered parsimonious. The RMSEA value was 0.041, which is less than the upper bound of 0.08; thus, a good fit of the model was achieved. In addition, the incremental fit indices were also within a reasonable range: IFI = 0.938, TLI = 0.935, and CFI = 0.938; all of these exceeded the recommended value of 0.90. The GFI and AGFI values were 0.881 and 0.869, respectively, and both exceeded the acceptable threshold of 0.80. Overall, the above results show that the structural equation model achieved an acceptable-to-good level of fit, and the proposed model was suitable for testing the hypothesized relationships among perceived destination image, electronic word of mouth, perceived destination value, visit motivation, visit attitude, visit intention, and visit behavior.

**Table 8:** SEM model fit summary.

Assessment	χ2/df	RMSEA	GFI	AGFI	IFI	TLI	CFI
Recommended threshold	<3	<0.08	>0.8	>0.8	>0.9	>0.9	>0.9
Observed value	2.154	0.041	0.881	0.869	0.938	0.935	0.938

**Figure 2:**
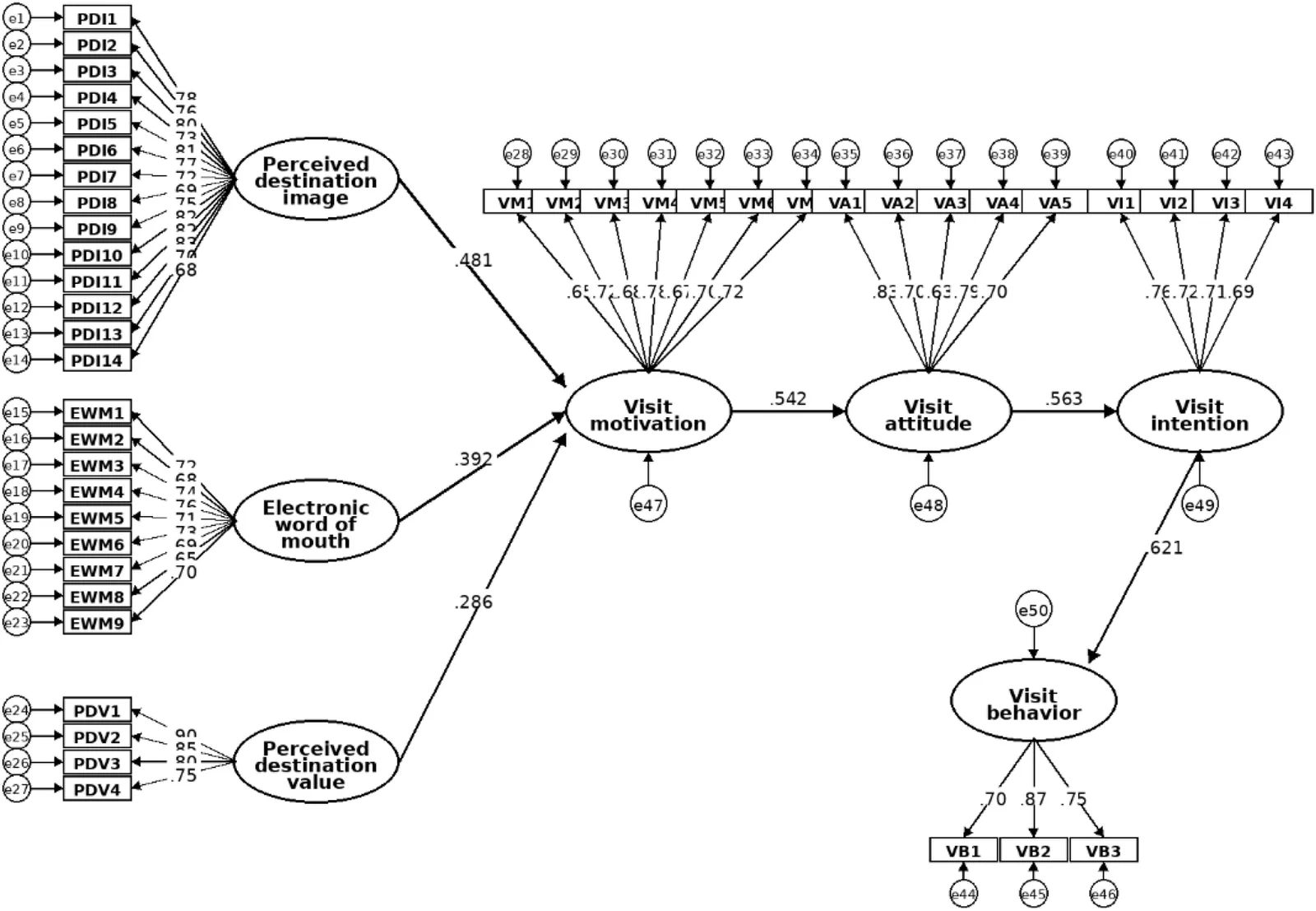
Structural model diagram. PDI, perceived destination image; Ewom, electronic word-of-mouth; PDV, perceived destination value; VM, visit motivation; VA, visit attitude; VI, visit intention; VB, visit behavior. All coefficients are standardized and significant at *p* < 0.001.

Figure 2 shows the structure of the latent variable model for testing the seven latent variables in this study. Based on the model, perceived destination image, electronic word-of-mouth, and perceived destination value are all positive predictors of visit motivation. Visit motivation positively affects visit attitude; visit attitude, in turn, influences visit intention; and finally, visit intention leads to visiting behavior. The standardised path coefficients in the model show that all the proposed paths are positive, and visit intention has the strongest direct effect on visit behavior. The retained measurement items also have reasonable factor loadings for their corresponding latent constructs and are therefore deemed to adequately represent the theoretical variables. Generally speaking, the model is a sequential mechanism that converts the perception of mainland Chinese tourists about Macao and online information evaluation and value assessment into visit behavior via motivation, attitude, and intention.

Table 9 shows the direct-path test results of the structural equation model. All six of the proposed direct effects were statistically significant at the 0.001 level in the results. Perceived destination image positively and significantly influenced visit motivation (*β*=0.528, C.R. = 12.313, *p* < 0.001); that is, tourists were more willing to visit after learning that Macao had a positive image. Electronic word-of-mouth was also positively correlated with the motivation to visit (*β* = 0.424, C.R. = 10.457, *p* < 0.001); that is to say, trustworthy and practical online tourism information enhanced tourists' desire to visit. Perceived destination value also had a positive impact on the motivation to visit (*β* = 0.263, C.R. = 7.688, *p* < 0.001), but this effect was weaker than that of destination image and electronic word of mouth. In the following behavioral path, visit motivation positively influences visit attitude (*β* = 0.517, C.R. = 11.397, *p* < 0.001), visit attitude positively affects visit intention (*β* = 0.602, C.R. = 13.145, *p* < 0.001), and visit intention positively drives visit behavior (*β* = 0.682, C.R. = 13.009, *p* < 0.001). Among the three antecedents of visit motivation, perceived destination image had the strongest standardized effect, followed by electronic word-of-mouth and perceived destination value. This indicates that mainland Chinese tourists' motivation to visit Macao was most strongly associated with their overall image of Macao, while online information and value evaluation also played significant motivational roles. The strongest direct path in the model was visit intention to visit behaviour, indicating that once tourists formed a clear intention to visit Macao, they were more likely to report actual destination-related behaviors. At the same time, the significant paths from motivation to attitude and from attitude to intention confirm the sequential psychological process proposed in this study.

**Table 9:** Structure equation model path test results.

Hypothesis	Path	Unstandardized Estimate (B)	S.E.	C.R.	p	Standardized Estimate (*β*)
H1	Perceived destination image → Visit motivation	0.528	0.041	12.313	***	0.481
H2	Electronic word of mouth → Visit motivation	0.424	0.057	10.457	***	0.392
H3	Perceived destination value → Visit motivation	0.263	0.025	7.688	***	0.286
H4	Visit motivation → Visit attitude	0.517	0.045	11.397	***	0.542
H5	Visit attitude → Visit intention	0.602	0.054	13.145	***	0.563
H6	Visit intention → Visit behavior	0.682	0.043	13.009	***	0.621

B, unstandardised estimate; β, standardised estimate.

*** *p* < 0.001.

Table 10 shows the results of mediation analysis after bias-corrected bootstrapping. Based on the above results, all of the indirect effects were statistically significant as none of the 95% confidence intervals included zero. Specifically, motivation for travel significantly mediates the paths of perceived destination image and visit attitude [effect = 0.273, 95% CI (0.225, 0.319)], electronic word-of-mouth and visit attitude [effect = 0.219, 95% CI (0.176, 0.268)], and perceived destination value and visit attitude [effect = 0.136, 95% CI (0.092, 0.182)]. These findings indicate that destination image, online information, and value evaluation influenced tourists' visit attitude mainly through the activation of visit motivation. Visit attitude also significantly mediated the relationship between visit motivation and visit intention [effect = 0.312, 95% CI (0.258, 0.366)], and visit intention significantly mediated the connection between visit attitude and visit behaviour [effect = 0.411, 95% CI (0.344, 0.472)]. These results indicate that motivation alone does not directly represent behavioral readiness. Instead, motivation needs to be transformed into a favorable attitude, and attitude needs to be further transformed into intention before being reflected in visit behavior. In addition, the chain mediation effects of visit motivation, visit attitude and visit intention were also significant in the relationships among perceived destination image and visit behavior [effect = 0.112, 95% CI (0.086, 0.144)], electronic word-of-mouth and visit behavior [effect = 0.090, 95% CI (0.067, 0.122)], and perceived destination value and visit behavior [effect = 0.056, 95% CI (0.037, 0.080)]. Among the three sequential mediation effects, the indirect effect of perceived destination image was the strongest, followed by electronic word-of-mouth and perceived destination value. This suggests that the behavioral conversion of mainland Chinese tourists visiting Macao is most strongly initiated by destination image, while digital information and value evaluation also contribute to the same sequential process. Overall, the mediation results support H7a–H10c and confirm that visit behavior is formed through a staged psychological mechanism rather than a single direct effect.

**Table 10:** Mediation effect analysis.

Hypothesis	Path	Standardized Indirect Effect	Boot SE	95% CI Lower	95% CI Upper	Result
H7a	Perceived destination image → Visit motivation → Visit attitude	0.273	0.024	0.225	0.319	Supported
H7b	Electronic word of mouth → Visit motivation → Visit attitude	0.219	0.023	0.176	0.268	Supported
H7c	Perceived destination value → Visit motivation → Visit attitude	0.136	0.023	0.092	0.182	Supported
H8	Visit motivation → Visit attitude → Visit intention	0.312	0.028	0.258	0.366	Supported
H9	Visit attitude → Visit intention → Visit behavior	0.411	0.033	0.344	0.472	Supported
H10a	Perceived destination image → Visit motivation → Visit attitude → Visit intention → Visit behavior	0.112	0.015	0.086	0.144	Supported
H10b	Electronic word of mouth → Visit motivation → Visit attitude → Visit intention → Visit behavior	0.09	0.014	0.067	0.122	Supported
H10c	Perceived destination value → Visit motivation → Visit attitude → Visit intention → Visit behavior	0.056	0.011	0.037	0.08	Supported

Bootstrap samples = 5,000. Bias-corrected 95% confidence intervals are used for the intervals.

## 5 Discussion

### 5.1 Main results

This study investigated the path by which mainland Chinese tourists' perceptions of Macao lead to a desire to visit. Rather than presenting tourist behavior as the direct result of isolated destination-related factors, the findings indicate a sequence of behavioral conversion in which the perceived destination image, electronic word-of-mouth and perceived destination value first activate the visit motivation, which then develops into a visit attitude, visit intention and finally visit behavior. This finding suggests that tourist behavior toward Macao is not formed immediately after tourists receive destination information or develop a favorable impression. Instead, it emerges through a staged psychological process in which tourists first evaluate the destination, then develop motivation, form an attitude, establish intention, and finally engage in destination-related behavior.

First, the research shows that perceived destination image and perceived destination value all positively influenced visit motivation. Among these three antecedents, perceived destination image showed the strongest standardized effect on visit motivation. This indicates that mainland Chinese tourists' motivation to visit Macao is most strongly associated with their overall perception of Macao's attractiveness. In the Macao context, destination image includes not only gaming and entertainment, but also cultural heritage, gastronomy, shopping, festivals, leisure atmosphere, urban scenery, and integrated tourism facilities. This finding is consistent with previous studies showing that destination image is closely related to tourists' motivation, attitude, satisfaction, loyalty, and behavioral intention (17, 28). It is also consistent with Wong and Lai's (2021) study on Macao, which found that non-gaming memorable tourism experiences are important for shaping destination image and behavioral intentions. However, this study further shows that destination image does not merely influence intention or behavior directly; rather, it first operates through visit motivation. Macao has long been associated with gaming tourism, while the findings suggest that a broader and more diversified destination image can provide the initial psychological stimulus for mainland Chinese tourists' motivation to visit.

Electronic word-of-mouth also had a significant positive effect on visit motivation. This finding reflects the growing importance of digital information in mainland Chinese tourists' short-haul and cross-border travel decision-making. For Macao, tourists often use online reviews, Xiaohongshu, Douyin, WeChat, online travel agencies, travel forums, and user-generated travel notes to evaluate transportation convenience, border-crossing arrangements, route planning, prices, crowding, food, shopping, attractions, and service quality before travel. Duong and Tung (41) found that eWOM can influence travel intention by shaping both attitude and motivation, while Filieri et al. (9) further showed that visual and verbal eWOM cues affect tourists' intention and decision to visit destinations and attractions. In the post-pandemic tourism environment, this role becomes even more important because travelers are more sensitive to uncertainty, risk, service reliability, and destination value. Nilashi et al. (23) also showed that eWOM in social network sites supports travelers' trust and decision-making during the COVID-19 period. Therefore, the significant effect of eWOM in this study can be explained by the fact that online information does not only transmit destination knowledge, but also provides external confirmation that Macao is convenient, enjoyable, safe, and worth visiting.

Perceived destination value had a positive effect on visit motivation, although its standardized effect was weaker than those of perceived destination image and eWOM. This result suggests that value evaluation is important, but it may not be the first psychological trigger for mainland Chinese tourists visiting Macao. For many mainland Chinese tourists, Macao is a nearby cross-border destination with strong symbolic, cultural, leisure, and entertainment meanings. Tourists may first be attracted by the city's image and online visibility, and then evaluate whether the trip is worth the money, time, and effort. This result is consistent with perceived value research, which argues that tourists' attitudes and behavioral responses are influenced by whether the perceived benefits of a destination justify the monetary, time, effort, and emotional costs involved (44). In the Macao context, perceived value is especially relevant for short-stay tourists because they need to judge whether a one-day or two-day trip can provide sufficient cultural, leisure, shopping, dining, and entertainment returns. Thus, perceived destination value strengthens visit motivation, but its role is likely to work together with destination image and eWOM rather than replace them.

Second, the results show that motivation, attitude and intention are the main psychological links in the chain of visiting behavior. Visit motivation positively impacts visit attitude, visit attitude positively influences visit intention, and visit intention positively drives visit behavior. This result is in line with the push-pull motivation theory, and the reasons for people travelling are due to both internal and external factors that motivate them to choose a specific destination (24) Based on the Theory of Planned Behavior, attitude is regarded as an indicator that determines whether one intends to act, and intention is near enough to behavior to be predicted directly (26). Recently, in their study of tourism, Pereira and others have also found that travel motivation, tourist attitude and the desire to travel are all related parts of people's destination selection process (45). This study extends these studies by integrating motivation, attitude, and intention into one sequential chain rather than treating them as separate predictors. For mainland Chinese tourists visiting Macao, motivation generated by cultural heritage, food, shopping, leisure, entertainment, and short-trip convenience needs to be translated into a favorable evaluation of visiting Macao. Only when this attitude becomes sufficiently positive does it strengthen visit intention and support actual destination-related behavior.

Thirdly, the main contribution of this paper is to explain the intention-behaviour link. Existing research on tourism generally focuses on travel intention or revisit intention, and these are not the same as actual behaviour. Xu and Su (46) pointed out that a tourist's desire to buy may not be realized in practice because the two are driven by different reasons. Recently, studies on sustainable tourism have also pointed out that behavioral intention should not be confused with actual behaviour; that is to say, some tourists may express a desire for sustainability without taking any corresponding actions (37). Wut et al. (2) further argued that attitude to behavior and intention to behavior gaps remain important theoretical issues in tourism research. This study responds to this gap by using visit behavior as the final outcome rather than stopping at visit intention.

The chain mediation results show that perceived destination image, electronic word-of-mouth, and perceived destination value all influenced visit behavior indirectly through visit motivation, visit attitude, and visit intention. This finding indicates that mainland Chinese tourists' visit behavior in Macao is not the immediate result of destination image, online information, or perceived value. Rather, these front-end perceptions need to pass through motivational activation, attitudinal evaluation, and behavioral readiness before being reflected in visit behavior. Among the sequential indirect effects, the pathway beginning with perceived destination image was the strongest, followed by electronic word-of-mouth and perceived destination value. This pattern suggests that Macao's behavioral conversion process is first image-driven, then information-supported, and finally value-reinforced.

In general, the findings respond to the two research objectives of this study. For the first objective, the results confirm that perceived destination image, electronic word-of-mouth, and perceived destination value are important antecedents of mainland Chinese tourists' visit behavior, but their influence is mainly transmitted through motivational activation and subsequent psychological links rather than through a simple direct route. For the second objective, the results confirm the mediating roles of visit motivation, visit attitude, and visit intention in the relationship between destination-related perceptions and visit behavior. Compared with previous studies that examined destination image, eWOM, perceived value, motivation, attitude, intention, and behavior separately, this study integrates these constructs into a sequential behavioral conversion model. Therefore, the main value of the findings lies in explaining not only whether mainland Chinese tourists want to visit Macao, but also the staged psychological process through which their perceptions are transformed into behavior.

### 5.2 Theoretical contributions

This study makes three main theoretical contributions to tourism behavior research. First, it extends tourism behavior research from intention-centered explanations to behavior-oriented explanations. Most of the existing tourism research has focused on travel intention, revisit intention or recommendation intention as the final outcome, and behaviour has received relatively little attention. However, intention is not equivalent to behavior. Previous studies have indicated that tourists' intentions are not always reflected in subsequent tourism-related actions, such as travel, consumption, or destination participation (47). This study responds to this limitation by using visit behavior as the final dependent variable and examining the psychological process through which mainland Chinese tourists' perceptions of Macao are transformed into behavioral engagement. Therefore, the contribution of this study is not only to explain why tourists intend to visit Macao, but also to clarify the mechanism through which favorable perceptions are converted into visit behavior.

Second, this study proposes and empirically tests a sequential perception, motivation, attitude, intention, and behavior mechanism. Previous tourism studies have shown that destination image, perceived value, electronic word-of-mouth, motivation, attitude, and intention are related to tourists' destination choices, but these variables have often been examined separately or through partial relationships. For example, destination image and perceived value have been linked with satisfaction, loyalty, and behavioral intention (48), and eWOM has been extensively studied in the context of destination image, attitude and travel intention (30). Compared with these studies, the research connects front-end destination-related perceptions, middle-stage psychological transformation, and back-end behavioral outcomes within one integrated sequential model. The results show that perceived destination image, eWOM, and perceived destination value first activate visit motivation; visit motivation then shapes visit attitude; visit attitude strengthens visit intention; and visit intention finally contributes to visit behavior. This sequential explanation advances tourism behavior research by showing that tourist behavior is not produced by a single direct factor, but by a staged psychological conversion process.

Third, this study enriches the theoretical application of the Theory of Planned Behavior by incorporating destination image, eWOM, perceived destination value, and visit motivation before attitude formation. The original Theory of Planned Behavior mainly explains behavior through the attitude-intention-behavior sequence, and tourism decision-making is also affected by external information and value evaluation before the attitude is formed (26, 49). Recently, some studies have shown that eWOM can change the image of a place in the minds of tourists and thus affect their travel emotions and travel intentions (10). Similarly, tourists have also listed perceived value as a reason for their satisfaction, loyalty and behavior intention; that is, they weigh the benefits of a place against the cost in terms of money, time and effort (19). By placing perceived destination image, eWOM, and perceived destination value at the front end of the model, this study demonstrates that tourists' behavioral decisions are affected by pre-attitudinal appraisals of destination attractiveness, online information credibility, and cost-benefit value. More importantly, by positioning visit motivation between these appraisals and visit attitude, the study clarifies how Push-Pull Theory interfaces with TPB. Destination image, eWOM, and perceived value explain what tourists evaluate before travel; Push-Pull Theory explains why these evaluations activate motivation; and TPB explains how motivation develops into attitude, intention, and behavior.

Overall, the theoretical contribution of this study lies in constructing an integrated behavioral conversion framework rather than simply adding more variables to an existing model. The findings suggest that mainland Chinese tourists' visit behavior toward Macao should be understood as a sequential process in which destination perception, digital information, and value evaluation stimulate motivation, motivation shapes attitude, attitude strengthens intention, and intention supports behavior. This framework provides a more complete explanation of tourist behavior formation and may help future tourism studies move beyond single-factor models and intention-only explanations.

### 5.3 Practical applications

The findings provide practical implications for different Macao tourism stakeholders, including tourism authorities, destination marketers, tourism enterprises, and public service departments. First, Macao tourism authorities and destination management organizations should strengthen the diversified and non-gaming image of Macao. Since perceived destination image significantly stimulates the desire to visit, Macao should be promoted not only as a gaming and entertainment destination, but also as a cultural, gastronomic, leisure, family-friendly, and short-break destination. The Macao Government Tourism Office and related destination management organizations can design specific theme-based routes for mainland Chinese tourists, such as heritage walking routes, gastronomy routes, festival routes, city-walk routes, family leisure routes, and night tourism routes. These routes can connect the Historic Centre of Macao, local food streets, cultural performances, museums, shopping areas, waterfront leisure spaces, and festival activities. For Guangdong tourists, one-day and weekend routes can be emphasized because of geographical proximity. For tourists from Shanghai, Zhejiang, Jiangsu, Beijing, Sichuan, and other longer-distance source markets, two-day or three-day cultural, shopping, leisure, and entertainment packages can be promoted. These actions can help mainland Chinese tourists form a broader and more favorable image of Macao and further stimulate visit motivation.

Second, destination marketers, online travel platforms, and digital communication teams should improve the credibility, usefulness, and completeness of Macao-related eWOM. A large number of eWOM will motivate tourists to visit, and thus shape their expectations before a trip. Therefore, Macao tourism stakeholders should manage destination-related content on platforms such as Xiaohongshu, Douyin, WeChat, online travel agencies, travel forums and review websites more strategically. Official accounts and tourism platforms should provide updated and practical information about border-crossing procedures, transportation options, route planning, ticket prices, opening hours, food recommendations, cultural activities, shopping areas, family-friendly attractions, crowding levels, and recommended visiting times. In addition, tourism authorities and businesses can cooperate with credible travel bloggers, local residents, and previous tourists to produce experience-based content, such as short videos, itinerary guides, budget plans, and real visitor reviews. Negative eWOM should also be monitored and addressed. Complaints about crowding, high prices, poor service quality, unclear routes, or low value for money should be responded to through both online clarification and offline service improvement.

Third, hotels, restaurants, shopping malls, travel agencies, entertainment enterprises, and integrated resorts should improve perceived destination value, especially for short-stay mainland Chinese tourists. The positive effect of perceived destination value on visit motivation suggests that tourists are more likely to travel to Macao if they believe that their trip will be worthwhile in terms of money, time, effort and experience. Tourism enterprises can jointly develop one-day and two-day value packages that combine heritage sites, local food, shopping discounts, cultural performances, leisure activities, family-friendly attractions, and night tourism experiences. These products should have transparent prices, convenient booking processes, clear route arrangements, and flexible combinations for different tourist groups. For example, hotels can cooperate with restaurants, cultural attractions, and transportation providers to offer bundled products, while integrated resorts can connect entertainment facilities with non-gaming cultural and leisure experiences. These measures can help tourists perceive Macao as a high-value short-haul destination rather than only a high-consumption entertainment city.

Fourth, transportation departments, public service providers, and urban managers should reduce practical barriers between visit intention and visit behavior. The results show that intention is a direct cause of the will to travel, but this intention will only translate into actual travel behaviour when conditions are favourable and reliable. Therefore, Macao needs to enhance the information on cross-border transportation, route planning, multilingual signage, mobile navigation, crowd-flow guidance, accessibility of public facilities. For short-stay mainland Chinese tourists, time efficiency is especially important. Clear information about border-crossing time, bus routes, walking routes, attraction distances, crowding conditions, and alternative itineraries can reduce travel uncertainty and make it easier for tourists to transform intention into actual visit behavior. In crowded areas and major transport hubs, real-time crowd guidance and route recommendations should be strengthened to improve tourists' travel experience.

In summary, Macao tourism stakeholders should not treat destination image building, online communication, perceived value enhancement, and mobility support as separate management issues. The findings suggest that these elements jointly contribute to the psychological conversion from destination perception to visit motivation, attitude, intention, and behavior. Therefore, Macao should adopt an integrated destination management strategy that combines branding, digital content management, tourism product design, service quality improvement, and transportation facilitation. This coordinated approach can help mainland Chinese tourists move from a favorable impression of Macao to stronger motivation, more positive attitude, clearer travel intention, and actual visit behavior.

### 5.4 Limitations and future research

This study has several limitations that should be considered when interpreting the findings. First, this study used a quota-based convenience sampling approach and focused only on mainland Chinese tourists who had visited Macao. Although regional quotas and screening questions were used to improve sample relevance and coverage, the sample was still collected through accessible online and offline channels rather than probability sampling. Therefore, the findings should be generalized with caution. Future research may cooperate with tourism authorities, hotels, border checkpoints, transport stations, travel agencies, and online travel platforms to obtain more representative data from different tourist groups.

Second, this study adopted a cross-sectional survey design. The data captured respondents' perceived destination image, electronic word-of-mouth, perceived destination value, visit motivation, visit attitude, visit intention, and visit behavior at one point in time. Therefore, the results can explain the associations among these variables, but they cannot fully confirm the causal development of tourists' psychological changes across different travel stages. Future research may use longitudinal designs to track tourists before, during, and after their visits to Macao. This would help clarify whether destination perceptions before travel are gradually transformed into motivation, attitude, intention, and behavior over time.

Third, although this study extended the outcome variable from visit intention to visit behavior, visit behavior was measured through self-reported questionnaire items rather than objective behavioral records. Self-reported behavior may be influenced by memory bias, social desirability, and respondents' subjective interpretation of their travel experiences. In the future, in combination with booking records and other sources of data such as mobile location information, transaction data, online reviews, social media check-ins and platform-based behavior traces from surveys, more objective and accurate measurements of tourist behavior will be carried out.

Fourth, the proposed model focuses on a linear sequential mechanism from destination-related perceptions to visit behavior through motivation, attitude, and intention. Although this model was supported by the empirical results, tourist behavior in Macao may also vary across different visitor segments. This study did not further distinguish between first-time and repeat visitors, same-day and overnight tourists, gaming-oriented and non-gaming tourists, or tourists from nearby Greater Bay Area cities and more distant mainland regions. Future research may add more moderators in the study of travel behaviour, such as travel frequency, income level, age group, companions on trips, destination familiarity, perceived risk, cultural identity or length of stay, to explore differences in behavioral conversion across various tourist groups. These factors may explain why some tourists convert favorable perceptions into actual visit behavior more strongly than others.

Finally, this study focuses on Macao, a compact, high-density, short-haul cross-border destination with a distinctive combination of Chinese and Western cultural heritage, integrated resorts, entertainment facilities, gastronomy, shopping, and a high proportion of mainland Chinese tourists. These contextual characteristics may limit the generalizability of the findings to other destinations. Future research may compare Macao with other short-haul or regional destinations for mainland Chinese tourists, such as Hong Kong, Singapore, Thailand, Japan, and South Korea. Comparative studies could further test whether the proposed perception–motivation–attitude–intention–behavior mechanism operates similarly across different destination environments.

## 6 Conclusion

This study examined the sequential psychological mechanism through which mainland Chinese tourists' destination-related perceptions are transformed into visit behaviour in Macao. Based on the 693 valid responses, tthe findings show that perceived destination image, electronic word-of-mouth, and perceived destination value positively influence visit motivation. Visit motivation further shapes visit attitude, visit attitude strengthens visit intention, and visit intention finally contributes to self-reported visit behavior. These results indicate that mainland Chinese tourists' behavior toward Macao is not produced directly by destination perception alone, but through a staged process of motivational activation, attitudinal evaluation, behavioral readiness, and behavioral expression.

Theoretically, this study contributes to tourism behavior research by moving beyond intention-centered explanations and proposing a sequential framework that links perception, motivation, attitude, intention, and behavior. By incorporating destination image, eWOM, and perceived destination value before motivation and attitude formation, the study clarifies the connection between external destination appraisals and internal psychological responses in tourist decision-making. In this sense, the study provides a more complete explanation of the process through which mainland Chinese tourists' favorable perceptions of Macao are converted into visit behavior.

Practically, the findings suggest that Macao tourism stakeholders should not rely only on general destination promotion or gaming-centered attraction. Instead, Macao should strengthen its diversified destination image, improve the credibility and usefulness of online tourism information, enhance perceived value for short-stay mainland Chinese tourists, and reduce practical barriers related to transportation, route planning, crowd management, and public services. These coordinated measures can help mainland Chinese tourists move from favorable destination perceptions to stronger motivation, positive attitude, clear visit intention, and actual visit behavior.

## Data Availability

The raw data supporting the conclusions of this article will be made available by the authors, without undue reservation.
